# Low-Energy Membrane Process for Concentration of Stick Water

**DOI:** 10.3390/membranes8020025

**Published:** 2018-05-27

**Authors:** Jinxiang Zhou, Scott M. Husson

**Affiliations:** 1 Department of Chemical and Biomolecular Engineering, Clemson University, 127 Earle Hall, Clemson, SC 29634, USA; jzhou@purilogics.com; 2 Animal Co-Products Research and Education Center, Clemson University, 250 Poole Agricultural Center, Clemson, SC 29634, USA

**Keywords:** dewatering, engineered osmosis, fouling, rendering, wastewater

## Abstract

This communication describes the application of forward osmosis (FO) to concentrate stick water, a nutrient-rich water byproduct of meat rendering operations. The objectives of the study were to carry out a set of batch FO runs in concentration mode to determine the maximum achievable stick water concentration and to perform a preliminary cost analysis for operating a FO/reverse osmosis membrane separation process for comparison to an evaporative concentration process. The study examined the roles of feed and draw solution stir rates, temperature, feed concentration, and draw solution ionic strength on flux using commercial cellulose triacetate membranes. Results show that FO could concentrate the stick water up to 45 wt %; however, concentrations above about 30 wt % would be difficult to process through conventional membrane configurations. Preliminary operating cost estimations show that the energy cost of the FO process is about 5.3% of the energy costs for a single-effect thermal evaporation process; and, assuming a 2-year membrane lifetime, the total operating cost using FO membranes was estimated to be about 23.1% of the operating cost using such a thermal evaporation process.

## 1. Introduction

Stick water is a byproduct of meat rendering facility operations that contains dissolved protein, minerals, residual oils, vitamins, and amines/ammonia [1]. Dewatering of stick water to recover edible protein conventionally is done by energy-intensive evaporation and drying operations (e.g., in a drum dryer or disc dryer) [1]. In these high temperature operations, some degradation of substances important to the nutritional value of the recovered protein will occur. Spray drying or spray granulation [2] may be an option, as the short residence times minimize degradation reactions. Still, initial dewatering is needed prior to the drying step. Beyond operational considerations, higher feed solids may also decrease off-flavor intensity for some protein products [3].

Membrane operations offer a low-temperature, low specific energy alternative for initial dewatering of stick water; however, an initial set of measurements found that pressure-driven membrane processes (microfiltration, ultrafiltration, reverse osmosis) were unsuccessful due to the high resistance caused by solids deposited on the membrane surface. Prior work has shown that this resistance increases with applied pressure because the solids layer is compressible [4]. Duke and coworkers [5] evaluated membrane distillation (MD) for recovery of water and reduction of stick water volumes. They demonstrated that MD could be used to produce process poultry, fish, and bovine stick waters; although, membrane fouling was a problem that will need to be addressed.

This study explored a low-temperature forward osmosis (FO) membrane alternative to evaporation for the initial dewatering of chicken feather stick water. Unlike pressure-driven membrane processes, FO uses a high ionic strength draw solution on the permeate side of the membrane to “draw” water from the feed to the permeate [6]. As a result, FO is capable of concentrating biomass [7,8] and offers excellent dewatering efficiency with less fouling potential compared to pressure driven membrane processes [9]. Systematic experiments were conducted to explore the impact of operating factors on the rate of water removal and the maximum achievable chicken feather stick water concentration during batch operation. These factors include draw solution ionic strength, the starting feed concentration, temperature, and the feed and draw solution stirring velocities. Operation cost estimations were made to compare FO and thermal evaporation processes. Positive results obtained with FO encouraged us to rapidly share this early-stage work with the membrane community.

## 2. Materials and Methods 

### 2.1. Materials

Commercial cellulose triacetate (CTA) FO membranes were provided by Hydration Technology Innovations, LLC (HTI; Albany, OR, USA). Stick water samples were provided by a local rendering facility and were stored in plastic containers at ~2 °C until filtration. Sodium chloride (99.5%) was used as received from Fisher Scientific. Deionized water was produced from distilled water that was passed through a Milli-Q water purification system (EMD-Millipore, Burlington, MA, USA)

### 2.2. Forward Osmosis Apparatus and Measurement Methods 

**Figure 1:**
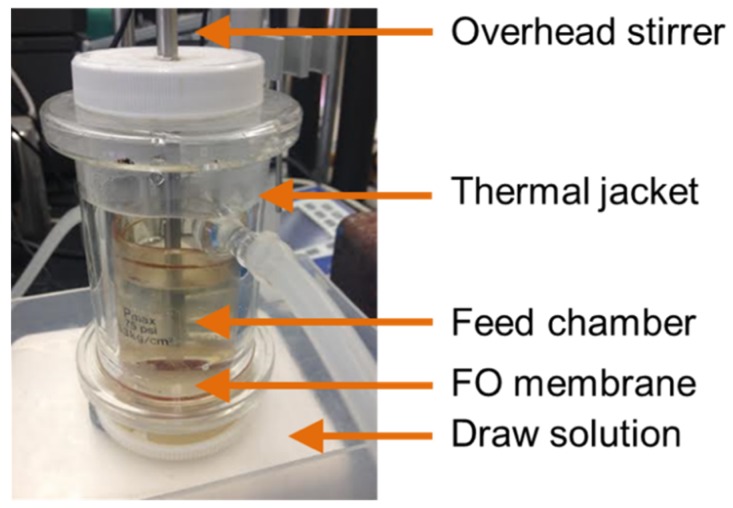
Forward osmosis apparatus for concentration of stick water.

An FO apparatus was designed and constructed for batch stick water concentration experiments. A 47-mm diameter membrane disk was placed in a modified Amicon stirred cell (Figure 1) that allows the membrane to have direct contact with the feed and draw solutions. The membrane was placed in the typical orientation for FO, with the draw solution placed against the support layer and the feed on the active layer [10]. The effective filtration area is 15.5 cm^2^. The upper chamber of the cell was filled with 25–50 g of stick water, and the cell was partially immersed into a 500 mL draw solution bath to keep the draw solution concentration nearly constant. Aqueous draw solutions from 0.10 to 1.50 M NaCl were studied. An overhead stirrer and a magnetic stir plate were used to stir the feed and draw solutions using stir speeds from 0 to 360 rpm. A thermal jacket was placed outside of the cell. Hot water was pumped through the thermal jacket to heat the cell and maintain a constant temperature from 25 to 75 °C. The draw solution was maintained at the same temperature as the feed.

During concentration experiments, the cell mass was measured and recorded at different times using a balance (OHAUS Explorer Precision). Stick water solids mass fraction (C_t_) was calculated using Equation (1):

(1) $$C_{t}=\frac{m_{0}^{\prime }}{m_{t}-m_{0}}{\;C}_{0}$$

m_t_ is the combined mass (g) of the cell and residual stick water, m_0_ is the mass (g) of the clean cell, $m_{0}^{\prime }$ is the initial mass (g) of stick water in the cell, and C_0_ is the initial mass fraction of solids in the stick water. Ct and C_0_ also were measured directly using a thermogravimetric analysis (TGA) instrument (TA Instruments SDT-Q600). 

Equation (2) was used to calculate average flux over short time intervals during batch concentration.

(2) $$J_{w}=600\;\times \frac{m_{t,\;t2}^{}-m_{t,\;t1}^{}\;}{\left(t_{2}-{\;t}_{1}\right)A}$$

J_w_ is the flux (L·m^−2^·h^−1^, LMH), t_i_ (min) is time point the measurement was taken, and A is the membrane area (cm^2^). Percentage water removal from the original stick water (R%) is an important indicator of the process efficiency. Equation (3) was used to measure R%:

(3) $$R\%=\left(1-\frac{\left(m_{t}-m_{0\;}\right)\times \;\left(1-C_{t}\right)\;}{m_{0}^{\prime }\;\times \;\left(1-C_{0}\right)}\right)\times 100\%$$

Relative flux was computed as the quotient of the flux at time t by the flux at time zero. Relative osmotic pressure difference was computed as the quotient of the osmotic pressure difference at time t by the osmotic pressure difference at time zero.

## 3. Results and Discussion

**Figure 2:**
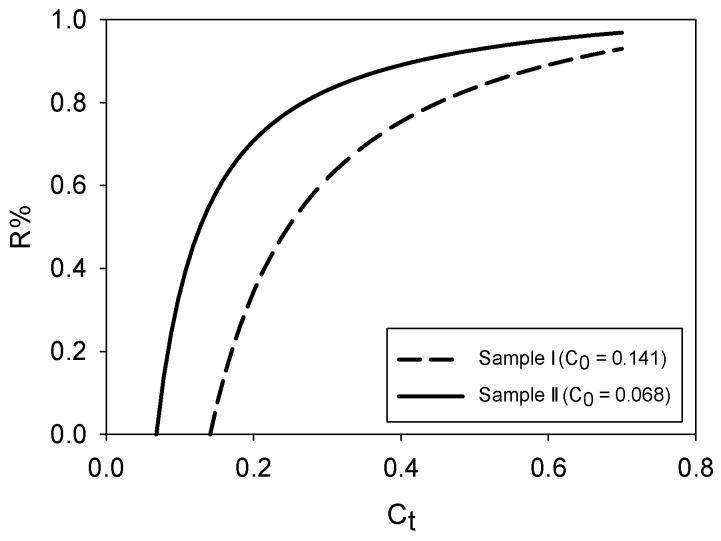
Required percentage water removal to concentrate stick water to target mass fraction solids, C_t_.

Like other wastewater streams generated by the rendering industry, stick water concentration varies temporally. In this study, two batches of stick water were received, each with different solids content: Sample I (14.1 wt %) and Sample II (6.7 wt %). Figure 2 presents the required percentage water removal to reach different solids mass fractions of stick water from Sample I and Sample II. Concentration from 14.1 to 30 wt % would require 62% of the water to be removed from Sample I. Over 82% of the water would have to be removed to reach 50 wt % solids, which was established by industrial members of the Animal Co-products Research and Education Center as the target solids content to enable spray drying. Concentration from 6.7 to 30 wt % solids would require 83% of the water to be removed from Sample II. Over 90% of the water would have to be removed to reach 50 wt % solids for this sample.

### 3.1. Impact of Draw Solution Concentration

**Figure 3:**
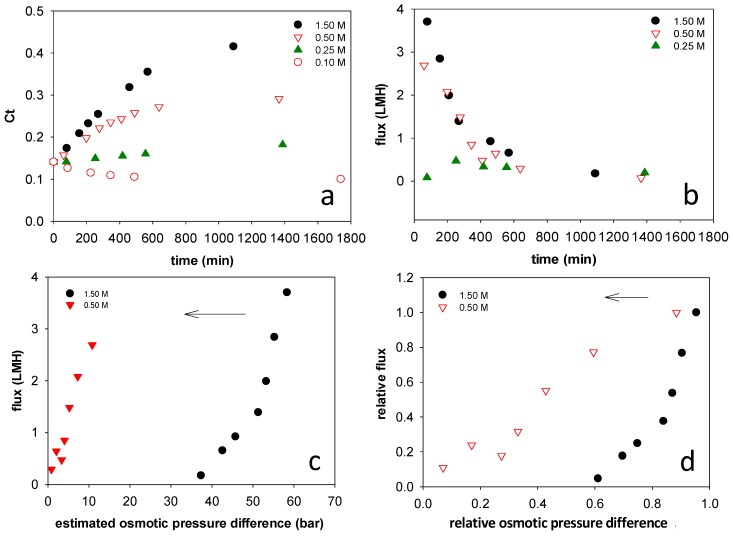
Impacts of draw solution concentration on stick water solid mass ratio and dewatering flux. Values in the legends indicate NaCl concentration in the draw solution. (**a**) Stick water mass fraction change with time using different draw solution concentrations; (**b**) Water removal flux change over time using different draw solution concentrations; (**c**) Water removal flux change versus estimated osmotic pressure difference across the membrane using different draw solution concentrations. (The arrow indicates the direction of change during dewatering); (**d**) Relative water removal flux versus relative osmotic pressure difference across the membrane using different draw solution concentrations. (The arrow indicates the direction of change during dewatering.).

Figure 3a shows the stick water solids mass fraction over time using different draw solutions. Data were collected using Sample I for these experiments. Both draw solution and feed solution stirring speeds were set to be 360 rpm. As is expected, higher concentration draw solutions were more effective at concentrating the stick water since FO is driven by the effective osmotic pressure difference across the membrane, as described by Equation (4) [11].

(4) $$J_{w}\;=\;A\left(\Delta \pi_{\mathrm{eff}}-\Delta P\right)$$

A is the intrinsic water permeability coefficient of the membrane; Δπ_eff_ is the effective osmotic pressure difference between the draw and feed solution accounting for the effects of internal concentration polarization, external concentration polarization, and reverse salt flux; and ΔP is the transmembrane hydraulic pressure difference. In our setup, the hydraulic pressure difference is only the pressure head associated with the column of feed within the cell. This value is 0.003 bar for our setup, compared to roughly 5 bar osmotic pressure for a 0.1 M draw solution, and therefore can be neglected.

In the case of 1.5 M NaCl draw solution, the stick water was concentrated to 41 wt %. In the case of 0.1 M NaCl draw solution, the stick water was diluted, indicating that the osmotic pressure of the stick water itself is higher than that of 0.1 M NaCl. From Equation (4), higher osmotic pressure on the feed side compared to the 0.1 M draw solution (Δπ_eff_ < 0) results in a reverse flow of water from the draw to feed solutions. The concentration increased only slightly using a 0.25 M NaCl draw solution.

Figure 3b presents water removal flux using draw solutions with different concentrations. As a basis for comparison, the starting flux value is lower than pure water flux of 6.03 LMH measured by Wang et al. [12] for the same membrane using a 0.5 M NaCl draw solution. Equation (4) explains this behavior since the stick water has a non-zero osmotic pressure. The flux decreased as the stick water concentration increased using 0.50 and 1.50 M draw solutions. The flux reduction could be attributed to two factors: (1) Increased concentration of the stick water reduced the osmosis pressure difference across the membrane. (2) A foulant layer developed on the membrane surface, which increased in thickness over time. The 1.50 M NaCl draw solution had the highest initial flux, but it also experienced the fastest flux reduction rate. Again, the flux was low using the 0.25 M NaCl draw solution suggesting that the effective osmotic pressure difference was low. Under these conditions, there appears to be nearly osmotic equilibrium between the feed and draw solutions. Osmotic equilibrium is a fundamental thermodynamic constraint on FO processes that limits the volume of water that can be recovered from the feed solution by a particular draw solution [13].

Assuming that feed side osmotic pressure increased linearly with concentration, Figure 3c replots the flux versus the osmotic pressure difference using the two draw solutions with the highest salt concentration. Using the initial flux and osmotic pressure difference as the reference, Figure 3d gives the relative flux change versus the relative osmotic pressure difference. Figure 3d enables comparisons of flux at equivalent relative osmotic driving forces using two different draw solutions. In Figure 3d, relative flux using 1.50 M NaCl draw solution decreased faster than that using 0.50 M NaCl. Furthermore, for the same relative osmotic driving force, the relative flux is lower when using 1.50 M NaCl draw solution. These results suggest that membrane fouling was more significant using higher concentration draw solution, as might be expected since higher initial flux rapidly brings more foulant material to the surface. A similar finding was presented by Zou et al. [14], who observed a critical flux behavior for FO operation.

### 3.2. Impact of Feed and Draw Solution Stirring Speeds

**Figure 4:**
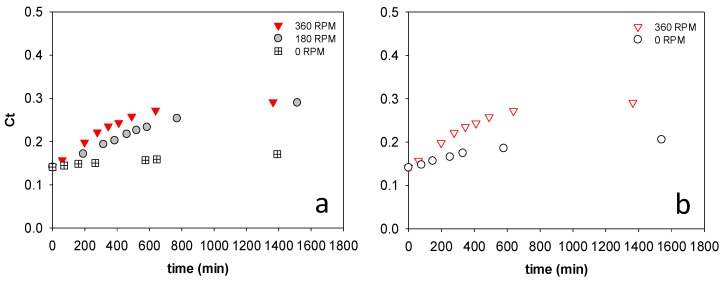
Stick water solids mass fraction change with time using (**a**) different feed side stir speeds and constant draw solution stir speed of 360 rpm and (**b**) different draw solution stir speeds and constant feed stir speed of 360 rpm.

Figure 4a,b show the impact of feed solution and draw solution stirring on the concentration process with Sample I. In both cases, a 0.5 M NaCl draw solution was used. Increasing the stirring speed on the feed and draw solution sides of the membrane from 0 to 360 rpm improved the initial flux. This can be explained by the disruption of the external concentration polarization (ECP) layer on the feed side and dilutive internal concentration polarization (ICP) on the draw solution side of the membrane with stirring [10]. While the rate of concentration was improved by increasing stirring speed, no difference in the final concentration was observed by increasing the stirring speed from 180 to 360 rpm. 

### 3.3. Impact of Stick Water Concentration

**Figure 5:**
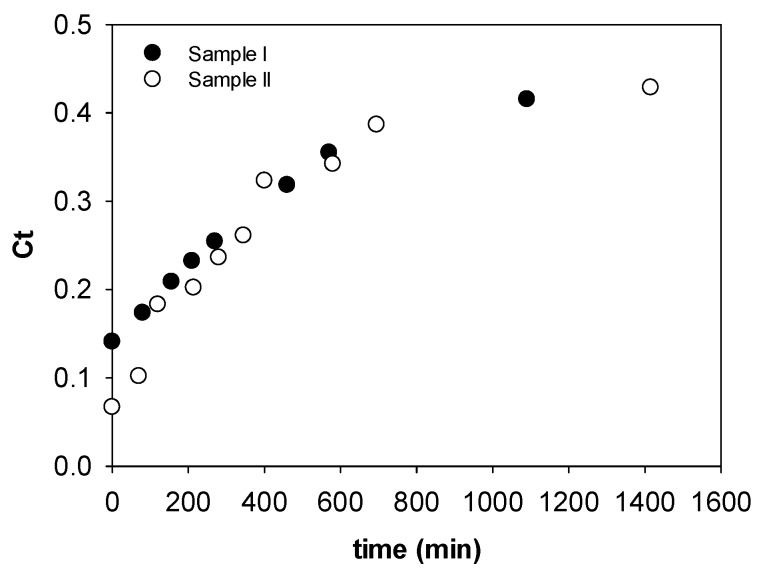
Impact of stick water mass fraction solids on the rate of concentration.

Figure 5 compares the concentration process for Samples I and II. Since the solids content of the samples differed, the volume of stick water charged to the cell was adjusted to keep the total solids content constant. Sample II, with a lower starting concentration, had a faster rate of concentration than Sample I, which is consistent with the fact that there is a higher osmotic driving force at the lower initial feed concentration; however, the rates eventually became the same at longer times when the concentration curves merged. One conclusion from this study is that the FO method is able to handle fluctuations in feed concentration, which is an advantage for treating rendering facility waters that change regularly depending on the input to the rendering facility. 

### 3.4. Impact of Temperature

**Figure 6:**
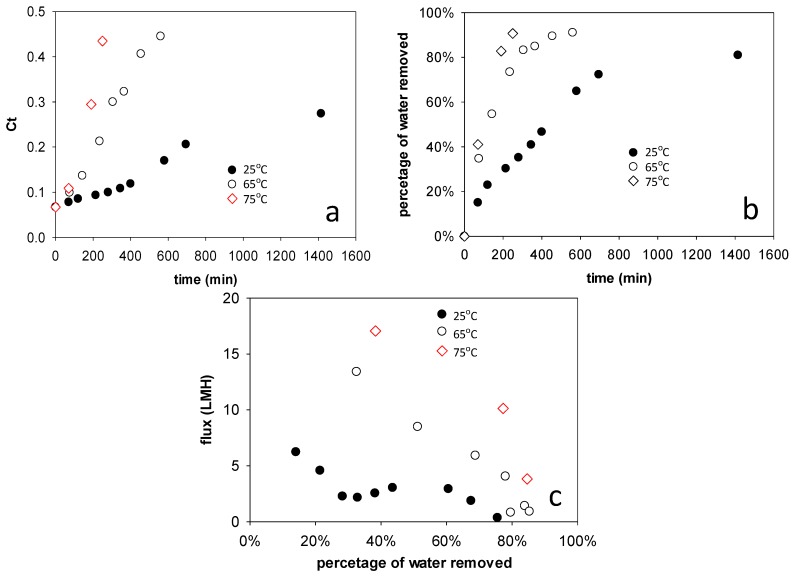
(**a**) Solid concentration of stick water over time at different temperatures; (**b**) Percentage of water removed over time at different temperatures; (**c**) Dewatering flux versus percentage of water removed at different temperatures. Starting concentration was 6.7 wt %.

Figure 6 shows the impacts of operating temperature on the stick water concentration process. This set of studies used 1.0 M NaCl solution as the draw solution. Stirring speeds of 360 rpm were applied on both feed and draw solution sides. Figure 6a shows the change of stick water solids mass fraction over time at elevated temperatures. For concentration studies at 65 and 75 °C, the maximum mass fraction attained was around 0.45, which was slightly higher than the maximum concentration attained at 25 °C. The concentrated stick water started to become a thick paste at around C_t_ = 0.30. Above this value, the stick water was difficult to remove from the membrane cell. Figure 6b shows the percentage of water removed from the original stick water using different temperatures. Whereas it took about 800 min to remove 80% of water at 25 °C, it took only about 230 min to remove 80% of water at 65 °C and about 150 min at 75 °C. After 70% water removal, the stick water was concentrated to C_t_ = 0.30. Figure 6c shows the flux change versus percentage water removed at different temperatures. For all the three temperatures, flux decreased with increasing stick water concentration. Similarly, the flux reduction can be attributed in part to increasing feed side ionic strength, which increases feed solution osmotic pressure and decreases the dewatering driving force. In addition, as water was removed from the feed side, a cake layer was observed on the FO membrane surface. The cake layer increases the transport resistance significantly. The dewatering flux was higher at higher temperature, which is expected since the intrinsic water permeability coefficient of the membrane (A in Equation (4)) increases as the viscosity of the solution decreases. While sample viscosities were not determined, pure water viscosities are well known (μ = 0.8903 cP at 25 °C, 0.4338 cP at 65 °C, and 0.3784 cP at 75 °C) [15]. Interestingly, after 80% water removal, the flux decreased by 69% at 25 °C, by 55% at 65 °C and by 35% at 75 °C. The lower flux reductions at higher temperatures may be explained by enhanced Brownian motion, which could reduce the foulant deposition.

### 3.5. Target Stick Water Concentration

The highest stick water solids mass fraction that can be achieved by FO appears to be about 0.45. However, as is shown in Figure 6, the water flux is low at mass fractions >0.30 (i.e., >80% water removal for Sample II). We also observed that the stick water becomes a paste above about C_t_ = 0.30. Thus, pumping the fluid through a flow cell or membrane module becomes problematic. 

Of note, 80% of the water was removed to achieve a stick water solids mass fraction of 0.30. Thus, a low percentage of additional water removal would be needed to attain a dry solids product. Given these considerations, one approach might be to concentrate stick water to C_t_ = 0.30 or lower using membrane FO, followed by thermal evaporation or other technologies (e.g., spray drying) to concentrate it to the target value. Based on the flux data collected, we estimated the membrane area that would be needed for a plant treating 24,480 kg/day stick water. Assumptions were that the original stick water has a concentration of 6.8 wt % and that the goal is to remove 80 wt % of the water. Table 1 reports the estimated minimum membrane area depending on the filtration temperature. This estimation did not consider membrane cleaning and replacement. At 75 °C, the required membrane area is only 30% of the area that is required at 25 °C. 

### 3.6. Operating Cost Estimation

A preliminary operating cost estimation was conducted based on the data collected in this study. Five key assumptions were used in this estimation:

(1) Plant output is 24,480 kg stick water per day.
(2) Stick water has a concentration of 6.8 wt % and the goal is to remove 80 wt % of the water either by FO-RO or triple-effect evaporation. No energy is required to heat the feed to 75 °C, as it is available directly from the cooking process at 90–95 °C [1].
(3) Membrane cost is $55/m^2^ for FO and $25/m^2^ for reverse osmosis (RO) membranes [16].
(4) Operating cost comprises only material cost and energy cost. For FO-RO, the material cost is assumed to be membrane cost only. For RO, a permeability of 2.8 L/m^2^·h·bar was assumed (Toray 70UB RO membrane), and a feed pressure of 60 bar was assumed. The osmotic pressure of the 1.0 M draw solution is 50 bar, leaving a 10-bar transmembrane pressure driving force to determine the membrane area needed. The energy cost is associated with pumping the feed and pumping costs to regenerate the 1.0 M draw solution using RO to remove water. Thus, FO functions as a pretreatment for RO [17]. This energy cost was determined following the steps outlined by Zhou et al. [18]. A pump efficiency of 60% was assumed. Cleaning was scheduled every 4 days and assumed to be 1 h long. Cleaning cost is usually 15–20% of the operating cost [19]. It was assumed to be 20% in this case. This amount was added to the final column of Table 2.
(5) Thermal evaporation is used as a comparison. Typical figures for steam consumption are 0.60 to 0.65 kg, 0.40 to 0.45 kg and 0.20 to 0.35 kg steam per kilogram water evaporated in double, triple and quadruple effect evaporators [1]. At 1 bar, it takes 2.257 MJ (0.6269 kWh) to generate one kilogram of steam. The approximate cost of power plant operating expenses for electric utilities using fossil steam is $0.036/kWh [20]. Based on these basic data, it should cost about $0.009 of fuel to evaporate one kilogram of water from stick water using triple-effect evaporation.

Table 2 summarizes the estimated cost break down using FO-RO membranes and triple-effect evaporation methods. The manufacturer suggests FO membrane lifetime to be 2 years [21]. Table 2 presents the operating cost assuming different membrane lifetimes. In an extreme case where membranes last only 3 months, the results indicate the total cost using FO-RO would be higher than using triple-effect evaporation. However, if the membrane lifetime is 2 years as suggested by the manufacturer, then the FO operating cost could be as low as 23.1% of the cost for evaporation. Moreover, the energy cost using FO-RO is only 5.3% of the boiling water evaporation method. 

## 4. Conclusions

This study evaluated a new, low-energy process for concentration of stick water to high solids levels. The FO operation is able to dewater stick water feed at low temperature (≤75 °C) to concentrations that would allow efficient spray drying. Thus, FO membranes could be used as the first dewatering step to remove the majority of water, followed by spray drying as a second unit operation to remove residual moisture. Based on preliminary operating cost estimations, this hybrid approach would have substantially lower cost than thermal evaporation alone and would avoid long-term contact of the recovered protein material with high temperatures that may degrade it.

## Figures and Tables

**Table 1:** Estimated minimum membrane area.

Stick Water Flow Rate (L·h^−1^)	Average Membrane Flux (L·m^−2^·h^−1^)	Membrane Area (m^2^)	Temp. (°C)
1020	13.5	76	75
1020	9.5	108	65
1020	4	256	25

**Table 2:** Estimated material and energy cost using FO-RO and triple-effect evaporation methods.

Frequency of Membrane Replacement	Membrane Cost ($)	Dry Material Weight (kg)	FO-RO Material Cost $/1000 kg Dry Material	FO-RO Energy Cost $/1000 kg Dry Material	Total FO-RO Cost $/1000 kg Dry Material *
3 months	4860	185,760	26.2	1.3	33.0
6 months	4860	371,520	13.1	1.3	17.3
12 months	4860	743,040	6.6	1.3	9.5
24 months	4860	1,486,080	3.3	1.3	5.6
Triple-effect evaporation cost	0	0	0	24.3	24.3

* Includes material, energy, and cleaning costs in the case of the membrane process.
